# mHealth App for Cannabis Users: Satisfaction and Perceived Usefulness

**DOI:** 10.3389/fpsyt.2015.00120

**Published:** 2015-08-27

**Authors:** Grégoire Monney, Louise Penzenstadler, Olivia Dupraz, Jean-François Etter, Yasser Khazaal

**Affiliations:** ^1^Department of Mental Health and Psychiatry, Geneva University Hospitals, Geneva, Switzerland; ^2^Faculty of Medicine, Institute of Global Health, University of Geneva, Geneva, Switzerland; ^3^Department of Psychiatry, University of Geneva, Geneva, Switzerland

**Keywords:** cannabis, addiction, app, mhealth, smartphone, cognitive behavior therapy

## Abstract

**Objective:**

The aim of this study was to describe the characteristics of cannabis users and their levels of satisfaction with Stop-cannabis, an app intended for cannabis users who want to stop or reduce their cannabis use or prevent relapse.

**Methods:**

A cross-sectional online survey was administered to users of Stop-cannabis, a French-language app for iOS and Android devices. All app users were invited to participate in the survey via a message sent to the app.

**Results:**

For hundred and eighty-two users answered the survey. The app was used daily by 348 of the participants (around 70%). More than 80% of participants (397) considered the app to have helped them “a little” or “a lot” to stop or reduce cannabis consumption. Most of the users’ suggestions were related to the number or the quality of the messages sent by, or displayed in, the app.

**Conclusion:**

This pilot study supports the feasibility of such an app and its perceived usefulness. A self-selection bias, however, limits the conclusions of the study. The efficacy of the app should be evaluated in a randomized controlled trial.

## Background

Cannabis is a widely used substance associated with harms, addiction, and possible psychiatric disorders in some users (1–8), including young adults and adolescents (9, 10).

During the last years, an increasing number of Internet-based self-help and treatments were developed as an attempt to give information, assessment, support, or treatment for people with substance use disorders or behavioral addictions (11–23).

Interventions, such as motivational interviewing and cognitive behavioral therapy, including those used in web-based treatment formats, have been shown to have a favorable impact on a number of lifestyle and health-related behavior (24, 25) and cannabis use (20, 26–28). Unfortunately, the number of users who seek help for cannabis addiction remains very low (29). This may be due to a perceived stigma and limited access to treatment (17, 30), or to expectations of care ineffectiveness (31). With the launch of mobile applications software (“apps”) and the wide dissemination of smartphones, however, a new opportunity for e-health and for the development of interactive tools has emerged (11, 32–35).

Particularly, apps combine mobile communication and computation in a handheld-sized tool, allowing new ways for clinicians to help people in real-time in order to promote positive change (36).

Several behavioral change techniques were considered by expert consensus as possibly useful for the design of apps aiming to help people with substance use disorders, such as self monitoring, goal setting, action planning, and feedback in relation to goals (37).

This potential recently led to the emergence of App-based technologies in order to help people with alcohol use disorders (37–40). Particularly (40), it was found that the smartphone delivered addiction-comprehensive health enhancement support system (A-CHESS) reduced the number of risky drinking days in comparison with treatment as usual controls in people with Alcohol use disorder. A-CHESS provides monitoring, information and real-time text messaging, communication and support.

Smartphone apps may therefore increase access to services and professional services for cannabis users who would otherwise not use them, or would use them only after the occurrence of serious consequences of their addiction. Furthermore, health-related smartphone apps allow people to integrate such technological support or “treatment” into their everyday lives, by way of ecological momentary assessments (EMAs) and ecological momentary interventions (EMIs) (41–43).

Nonetheless, several concerns have been reported in relation to such tools. In particular, several studies reported that the lack of concordance between the content of health-related apps available on the stores, including for smoking and substance use, and evidence-based recommended treatments, low emphasis on psychological needs related to the self-determination theory (i.e., low emphasis on autonomy, competence, and relatedness), and rarity of apps that include EMAs or EMIs (42, 44–47). Furthermore, an important gap still exists between the considerable number of apps for health available on the market and the limited number of scientific publications related to the field (48, 49).

One app for cannabis addiction has been previously described (50). This app was based on cognitive behavioral therapy and motivation enhancement therapy. Reactions of 10 cannabis users after a 2-h testing session (50) showed good overall satisfaction with the app by the participants. We recently developed a new app, Stop-cannabis, to help cannabis users stop or reduce their use of this substance.

The aim of this study is to describe the app^1^ and to describe the characteristics of cannabis users and their levels of satisfaction with the Stop-cannabis app. The app is intended for cannabis users who want to stop or reduce their cannabis use or prevent relapse.

## App Description

The app is associated with an Internet website^2^ and is available on Google Play (Android) and the App Store (iOS) at no charge without any commercial advertising or costly upgrading tools. The development of this app was funded by the local Health Department in Geneva, Switzerland. As in other self-help and web-based treatments for addictive disorders [Ref. (20, 42, 50–53), the Stop-cannabis app is based on screening and brief intervention (54–56), motivational interviewing (57)], and principles of relapse prevention in the treatment of addictive disorders (58, 59). Furthermore, and in accordance with self-determination theory (60), the app particularly emphasizes competence, relatedness, and autonomy (60), important features that should be included in all apps related to addictive behaviors (45). The App was described according to this conceptual framework as shown in Table 1.

**Table 1:** **Access to main app components and possible links with competence, relatedness, and autonomy**.

App components	Competence	Relatedness	Autonomy	Access
Social network	X	X		For all app users
EMA, EMI	X	X	X	After choosing a quitting date
Text messaging	X	X		After choosing a quitting date
App personalization			X	For all app users
Reinforcement after quitting	X		X	After choosing a quitting date
Brief intervention and feedback	X		X	For all app users
Psychoeducation	X			Via the website for all users
Motivational interview	X		X	Via the website for all users

Launched in February 2013, the app was downloaded by 13,734 users at the time of the study, involving around 700 active users/week (people with at least one section during a week) and 2000 active users/month (people with at least one active session during a month).

## App Components

### Brief intervention

**Figure 1:**
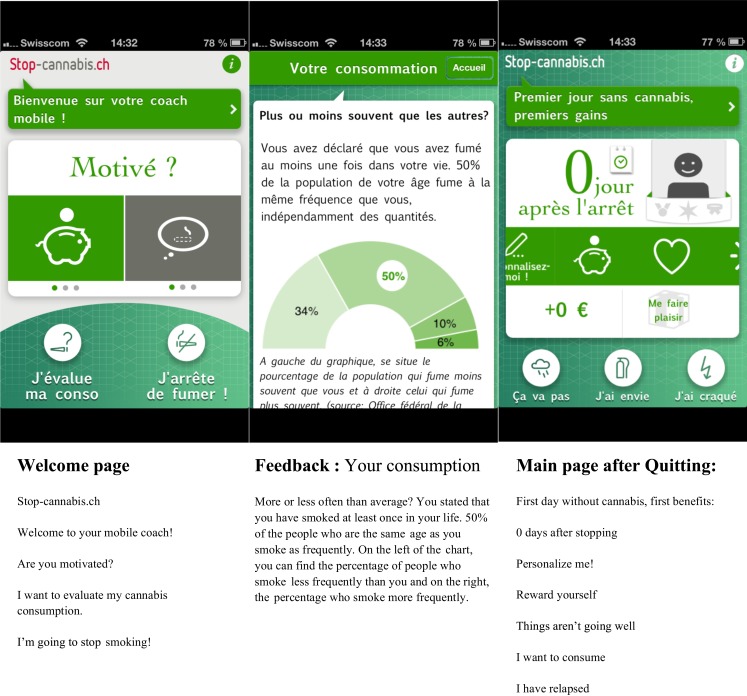
**Screenshots of the welcome page, the Feedback page, and the main screen with the benefits of change**.

The brief intervention is accessible to all users, without specific registration, and is based on a brief questionnaire on cannabis use followed by a set of brief individually tailored feedback messages (Figure 1). The questionnaire includes the French version of the cannabis-related questions from the Alcohol, Smoking and Substance Involvement Screening Test (ASSIST) (61–63). The feedback messages are written on the basis of each participant’s ASSIST scores. A feedback message is also given on the basis of the comparison between the frequency of reported cannabis use and the frequency of cannabis use by Swiss people from a similar age and sex group (percentage of people with less cannabis use or more cannabis use than the app user).

### Discussion forum

In the iOS app only, users can access a discussion forum called “La Tribu” (The Tribe), allowing written interactions with other users. Prior research suggests that this type of service is an important user demand (33). This discussion forum is moderated by a psychologist who also encourages discussions between users.

### Personalization of the app

To facilitate the appropriation of the tool, users can personalize the app with pictures. Such pictures may be related to personal motives to change (i.e., expected benefits, gifts, goals). These pictures can be seen the by user on the main screen at any time.

### Positive reinforcement

The main screen shows two metrics, the number of days since stopping cannabis use and the amount of money saved. Positive reinforcement is further offered in the form of awards that can be earned when reaching a given number of days since stopping cannabis use, a given number of times accessing the app, or a given number of times seeking advice.

### EMAs and EMIs

The app includes some patterns of EMAs and EMIs. Users can access automatic tailored advice or encouragement at the moment of exposure to potential relapse situations, such as craving, irritability, insomnia, appetite problems, and anxiety. He or she can also indicate if a lapse occurs: the app then delivers encouraging messages in order to reduce the risk of full relapse or the negative consequences of excessive guilt associated with lapse events.

### Text messaging

Automatized personalized text messages and emails that focus on helping users give up the consumption of cannabis are sent over a period of several months. Text messaging is activated upon the user’s selection of a cannabis quitting date.

### Association with an internet website^2^

In addition to the app tools, at the website, users can access additional forms of help, such as information pages (psychoeducation), automated online motivational interviewing, and addresses of local clinics.

## Materials and Methods

A cross-sectional online survey, in a specific format that is readable from smartphones, was posted on the Stop-cannabis website. A link to the survey was included in all app messages from February 2013 to December 2014 (up to twenty messages/users during this time-frame). After reading a description of the study on their smartphone, users accessed the online survey.

The study is part of the annual reports of quality monitoring of the app. This report is part of the requirements of the funding source (Canton of Geneva) and was approved by its board. Assessments and data were reported anonymously.

The questions were specifically designed for the survey and covered sociodemographic characteristics (gender, age), frequency of cannabis use, number of joints smoked daily before stopping (Table 2), frequency of the app use, and satisfaction with the app (Table 3). Participants also provided written comments on the app (Table 4). The ASSIST questionnaire is not included in the assessments of the study at hand. It is a part of the brief intervention offered to the app users.

**Table 2:** **User characteristics**.

Characteristic		*N* = 482
**Age (years)**		
	15–25	188 (39.0%)
	25–40	221 (45.9%)
	40–60	60 (12.4%)
	>60	0 (0%)
	No answer	13 (2.7%)
Male		
		335 (69.5%)
**Frequency of cannabis use**		
	Daily use	99 (20.5%)
	3–6 days/week	24 (4.9%)
	1–2 days/week	21 (4.35%)
	Several times a month	13 (2.7%)
	Less than once a month	11 (2.3%)
	I do not smoke anymore	307 (63.7%)
	I have never been a cannabis user	4 (0.82%)
	No answer	3 (1.87%)
**Number of joints smoked daily (mean ± SD)**		4.7 ± 2.6

**Table 3:** **Use and satisfaction relative to the application**.

**Frequency of application use, *N*(%)**		
	Several times/day	231 (46.2%)
	Once a day	117 (24.5%)
	Several times/week	59 (12.3%)
	Once a week	14 (2.9%)
	Several times/month	2 (0.42%)
	Once a month or less	0
	No answer	59 (12.3%)
**How helpful was the application to stop or reduce cannabis use?**		
	It did not help me at all	11 (2.3%)
	It helped me a little	187 (39.1%)
	It helped me a lot	216 (45.2%)
	No answer	68 (14.2%)
**How helpful were the messages (notifications)?**		
	They did not help me	11 (2.3%)
	They helped me	307 (64.2%)
	They helped me a lot	90 (18.9%)
	No answer	74 (15.5%)
**Global appreciation of the application**		
	Dissatisfactory	1 (0.2%)
	Not very satisfactory	12 (2.5%)
	Moderately satisfactory	26 (5.3%)
	Reasonably satisfactory	157 (32.6%)
	Very satisfactory	221 (45.9%)
	No answer	61 (12.8%)

*N = 478*.

**Table 4:** **User suggestions related to the app**.

Suggestions	*N* (%)
1. Increase the number of messages (more messages, longer duration)	37 (29.4%)
2. Improve existing messages	28 (22.2%)
3. Add more information, advice, and health-related information (e.g., health benefits, psychological effects)	10 (7.9%)
4. Do not change anything, the application is satisfactory as it is	8 (6.3%)
5. Refine the metrics (number of joints, possibility to restart at 0, personalization, etc.)	6 (4.8%)
6. Have more testimonies of former smokers	5 (3.9%)
7. Possibility to change the notifications setting	4 (3.2%)
8. Possibility to discuss with a professional	4 (3.2%)
9. Change visuals (e.g., color green)	4 (3.2%)
10. Increase the number of possible medals won	3 (2.4%)
11. Improve personalization tools	2 (1.6%)
12. Add the balance of the benefits and costs of cannabis use	2 (1.6%)
13. Keep a smoking (before quitting) and relapse history	2 (1.6%)
14. Add feedback on cannabis concentration in blood and urine	2 (1.6%)
15. Possibility to add more pictures and videos	2 (1.6%)
16. Suggest a drug consumption agenda (stopping gradually)	2 (1.6%)
17. Make the application for other OS (Android)	1 (0.8%)
18. Remove the Facebook option	1 (0.8%)
19. Simplify the ergonomics	1 (0.8%)
20. Improve the relaxation tool	1 (0.8%)
21. Improve the design	1 (0.8%)

After exclusion of incomplete answers and duplicate answers emerging from the same IP address, we performed a descriptive analysis of the answers. Free comments were grouped according to categories related to the idea expressed by the participants.

Descriptive statistics were used to summarize the participants’ characteristics and answers. Means and SDs as well as number and percent were reported.

## Results

### User characteristics

In total, 482 users aged 14–59 years were included in the analysis. Most participants (70%) were men, more than 40% were 15–25 years old, and 15% were under 20 years old. About 85% of the respondents were under 40 years old. Most participants (60%) were former cannabis users (described themselves as past cannabis users at the time of the survey). Daily cannabis use was reported by 20% of the participants, whereas about 30% reported at least weekly cannabis use and <1% had never smoked cannabis (Table 2). The users who never smoked cannabis were excluded from the analyses related to app’s use and satisfaction (Table 3). Former users smoked about 5 joints/day on average before quitting (minimum: 0; maximum: 9).

### User views on the app

The app was used at least weekly by most participants, daily by more than 70%, and several times a day by half of the participants (Table 3). About 80% of the users reported a good overall level of satisfaction with the app (“reasonably or very satisfactory”; Table 3). More than 80% of users considered the app to have helped them “a little” or “a lot” to stop or reduce cannabis consumption. Similarly, more than 80% of the participants found the app messages “helpful” or “very helpful” (Table 3).

We collected 190 free text comments posted by 150 users. Most of them, 126, were related to the app in general (Table 4) and 64 comments specifically related to the discussion forum “La Tribu” (Table 5). Half of the comments referred to the notification messages (Table 4, suggestions 1 and 2). Concerning these messages, several comments recommended improving the content of the messages (e.g., “more personalized”) and increasing the number of messages (especially after 4 months of abstinence, as the messages were least frequent in this period). The other suggestions were related to the following points: more personalization of tools (Table 4, suggestions 7, 9, 11, and 15); more information, more tailored feedback, and more rewards and training (Table 4, suggestions 3, 5, 10, 12, 14, and 20); and options related to the observation of one’s own cannabis use and advice on the reduction rather than the cessation of cannabis use (Table 4, suggestions 13 and 16). Only 3% of the suggestions were related to the possibility of speaking with a health professional.

**Table 5:** **Specific user suggestions related to the social network “La Tribu**.”

Suggestions	*N* (%)
1. Send a notification when someone adds a comment or a “like”	12 (18.70%)
2. Implement a private messaging option	11 (17.20%)
3. Possibility to write a longer message and to read it before publication	10 (14.10%)
4. Messages history	5 (7.80%)
5. More support and shared activities and photos between users	7 (7.80%)
6. Improve the moderation	4 (6.20%)
7. Arrange classification of messages by themes	3 (4.70%)
8. Increase the number of personalization options	3 (4.70%)
9. Possibility to block someone (i.e., less motivated persons)	2 (3.0%)
10. Public rewards such as medals	1 (1.50%)
11. Avoid group leading to non-inclusion of new members	1 (1.50%)
12. Publish information in relation to usage statistics	1 (1.50%)
13. Update the social network	1 (1.50%)
14. Easier registration	1 (1.50%)
15. Possibility to see if someone is online	1 (1.50%)

About 40% of the suggestions on the discussion forum were related to the improvement of the system used to manage messages and to options for mutual support between users (Table 5, suggestions 2, 3, 4, 5, and 7). Other suggestions were related to moderation of the forum, including the possibility of excluding a participant (Table 5, suggestions 6 and 9). The comments related to the social network mostly asked for improvement of “La Tribu” by adding tools similar to those used in other social networks, such as notification options or being able to see if a user is online.

## Discussion and Conclusion

### Principal results

This study suggests that the Stop-cannabis mobile app is acceptable and perceived as useful by the users. The app was appreciated by most of the participants and was furthermore considered helpful in either stopping or reducing cannabis use. More than 80% of users considered the app to have helped them “a little” or “a lot” to stop or reduce cannabis consumption. The study was not, however, designed to assess the effectiveness of the app and cannot provide an assessment of the effect of the app on cannabis use.

About 60% of respondents reported that they no longer smoked cannabis. However, it is unclear how many of them stopped smoking cannabis after using the app or whether they had stopped before downloading it, because we did not ask this question. About one-third of the user group comprised daily cannabis users and about one-third comprised weekly cannabis users. The usefulness of the app in current cannabis users is of high interest in consideration of the generally low rate of users among this group who seek medical or psychological help (64).

Most of the open comments by users were related to the text content of the app, asking for more personalized messages, longer support time, and more personalized content. Such aspects should be considered for future developments of this app. Perceived support from text messaging seems important for users and may be reinforced by support on the discussion forum. Most of the suggestions about the social network were also related to the text messaging options, highlighting the possible importance of this kind of support between users. Few open text comments asked for more interactions with health professionals, possibly because the users do not expect such support on the app.

## Limitations

It is likely that there was a self-selection bias in our study and that we enrolled a disproportionate number of users who were satisfied by the app or who were more involved in its use, as suggested by other studies on selection bias in Internet-based studies (65).

## Conclusion

As shown in other studies related to app for substance use (13, 40, 50, 66), «stop-cannabis»was appreciated by most of the participants and seen as useful. This is in accordance with the rapid spread of e-health (67–72).

The usefulness of the app in current cannabis users is of high interest in consideration of the generally low rate of users among this group who seek medical or psychological help (64).

Text messages were considered as supportive. Users however asked for more messages with more personalized contents. This is in concordance with other studies related to user’s views and preferences on text messaging (73, 74). Further studies linking messages content, the moment of delivery, interactive features, the level of tailoring and user satisfaction and preference may help to further improvements of such kind of interactions.

The app could be used for relapse prevention as well as a tool helping people who would like to prepare a quit attempt. Further studies in preparation may help to better understand how cannabis smokers use the app and what they expect from its use. Intra-app navigation analyses as well as further user’ satisfaction surveys would be helpful.

The app impact on cannabis use will be assessed. Naturalistic and randomized controlled trials linking clinical characteristics of the users with outcomes related to cannabis use are warranted.

Nevertheless, it seems that tools, such as the app presented here are probably useful for at least some cannabis users, possibly as a “primary intervention” in the community, as a screening tool to detect problematic cases, or as a complement to clinical interventions between medical visits.

Other studies are needed to assess the impact of such tools on cannabis use, as well as their possible use in different settings (i.e., as an adjunct to specialized treatments or general practitioner advice, or in non-clinical settings) or with different populations in terms of age, social status, or comorbid conditions.

Tools, such as the app presented here, are acceptable and probably useful for at least some cannabis users from the community.

## Conflict of Interest Statement

The authors declare that the research was conducted in the absence of any commercial or financial relationships that could be construed as a potential conflict of interest.

## Footnotes

^1^http://www.stop-cannabis.ch/les-app-gratuites-stop-cannabis-ch-pour-iphone-android

^2^http://www.stop-cannabis.ch
